# Utilization and Evaluation of Ethics Consultation Services in Neonatal Intensive Care

**DOI:** 10.3390/children11111349

**Published:** 2024-11-04

**Authors:** Pia Göbert, Pia von Blanckenburg, Rolf F. Maier, Carola Seifart

**Affiliations:** 1 Hospital for Pediatric and Adolescent Medicine, University Hospital of Giessen and Marburg, 35037 Marburg, Germany; 2 Ethics in Medicine Unit, Department of Human Medicine, Philipps University of Marburg, 35037 Marburg, Germany; 3 Department of Psychology, Clinical Psychology and Psychotherapy, Philipps University of Marburg, 35037 Marburg, Germany

**Keywords:** neonatology, ethics, neonatal intensive care unit, ethics consultation, field expertise

## Abstract

Background: The opportunities of perinatal medicine have improved, but this has also been accompanied by increasing ethical challenges. Clinical ethics consultation services (CEC) could support medical teams facing these. However, nothing is currently known about the availability, utilization and evaluation of CEC in German neonatology units. Methods: This study was designed as a national, descriptive, mixed quantitative–qualitative questionnaire study. The head physicians of the pediatric departments and the heads (medical and nursing) of the corresponding neonatal intensive care units of the 213 German perinatal centers were asked to participate. Results: Ninety percent of the respondents (responding rate 24.4–38.0%) stated that CEC are established and available. However, utilization is rather low [rarely N = 40 (54.1%), never N = 12, (16.2%), occasionally N = 19 (25.7%)], although it was rated as very helpful. There was a significant correlation between utilization and perceived general usefulness (r = 0.224, *p* = 0.033) and support (r = 0.41, *p* < 0.001); whereas evaluations differed significantly between professional groups (t = −2.298, *p* = 0.23, Cohen’s d = 0.42). Conclusions: The contradiction between the low utilization despite positive evaluations could be related to perceived hurdles. These and the different perceptions within the professional groups give rise to the consideration of whether alternative approaches, e.g., liaison services, would be preferable in neonatology.

## 1. Introduction

Over recent decades, the medical opportunities in neonatology have developed enormously. Although this has improved care, it also poses challenges, not least ethical ones. Indeed, the clinical care of premature infants, especially ones with a birth weight of less than 1250 g or with a gestational age of less than 29 + 0 weeks’ gestation, raises a number of ethical questions. Medical teams increasingly have to deal with difficult situations at the limits of viability in the neonatal intensive care unit (NICU). In addition to morally difficult situations connected, the team of the NICU is often exposed to the suffering of families, who are confronted with the illness or death of their (newborn) infant. These situations are, therefore, also emotionally stressful. That is why NICU or PICU teams suffer more from moral distress and burden than teams in other ICUs [1,2,3,4].

Ethical reflections have been an integral part of the practice of medicine since its origin, as they include social interaction processes as well as fundamental goods such as health and life. To support medical teams with ethical challenges in hospital care, clinical ethics consultation services (CEC) have been established as an important tool in recent decades [5]. Ethics (case) consultation could be defined as: “(…) a service provided by an individual consultant, team or committee to address the ethical issues involved in a specific clinical case” [6]. Its function is to provide a structured approach to identify, analyze and solve ethical issues in the daily clinical routine, for example, the question of treatment limitation or dealing with serious complications.

The organizational forms of ethics case interventions are diverse and range from ethics committees to flexible working groups. These structures offer various clinical ethics consultation services such as clinical ethics ward rounds, liaison services, individual case consultations and hospital ethics committees (HEC).

Ethics (case) consultation can promote interdisciplinary cooperation [7], improve communication within the team [8,9] and provide a platform for the discussion of different perspectives [10] with a higher probability for consensus for goals of medical care [9]. Additionally, some studies show positive effects such as higher satisfaction among staff [11,12], improved interprofessional collaboration [13,14] and a lower utilization of resources [15]. These advantages improve the quality of care which, in the end, is to the benefit of patients.

However, the studies mentioned refer almost exclusively to CEC in adult medicine. There is only rare information about ethics consultation in pediatrics available. But this would be very important because ethics consultation in pediatrics and neonatology differs from adult medicine in several ways. In neonatology—even more than in general pediatrics—patients are at the beginning of their lives and are, therefore, in a special situation. In addition, the regenerative capacity of premature and full-term babies is astonishing and the prognosis can, therefore, often only be determined with a lot of uncertainty. It is also not possible to speak to the patient themselves or to obtain indications of a presumed will. Furthermore, the legal guardians must be advised and supported accordingly, taking into account the individual circumstances. Thus, in Neonatology, the decision-making process is extremely difficult for infants with uncertain prognosis and, therefore, the question of ethical dilemma in neonatology and their solution requires special attention [16].

Nevertheless, there is only little research that deals specifically with the issue of CEC in neonatology. Thomas et al. analyzed ethics consultations with patients under the age of 18 from 2005 to 2013 [17] and showed that these were mostly requested by NICUs and focused on end-of-life issues. When analyzing 359 deaths in Dutch NICUs with a focus on ethical challenges, Verhagen et al. concluded that treatment limitation decisions were often made due to a lack of quality-of-life outcome expectations [18,19]. In a case series dealing with ethics consultations in terms of satisfaction and usefulness for families and medical staff (physicians in reproductive medicine, obstetrics and neonatology), the treatment teams seemed to be supported in their decision making by ethical reflection [12]. This is encouraged by a theoretical analysis concluding that establishing ethics frameworks in NICUs in particular, such as CEC and education in ethics, to support decision making is helpful and necessary [16,20].

There are studies that analyze the implementation and structure of CEC in German hospitals in general and demonstrate that CEC are widely established [21,22,23,24,25]. However, it is unknown to what extent this also applies to perinatal centers (PNC) and NICUs. The same applies to the question of how ethical consultation is assessed in neonatology and what barriers exist to its use.

Therefore, this study aimed to explore the implementation of ethics consultation services, their utilization, main topics and their evaluation by the medical teams in neonatology in Germany.

## 2. Materials and Methods

This study was designed as a national, descriptive, mixed quantitative–qualitative questionnaire study. The addressees of the questionnaire were potential users of ethics consultation services in neonatology in Germany.

Therefore, this study was conducted in the perinatal centers (PNC). PNCs have been established in Germany for the specific care of pregnant women and premature or full-term babies with special risks. Two questionnaires (A and B) were developed by an interdisciplinary team of neonatologist (RM, PG), clinical ethicists (CS, PG) and psychologist (PvB). The questions were formulated as closed, mixed or open questions, depending on what was being targeted. The descriptive part and the one part on the evaluation of clinical ethics consultation were mainly surveyed using closed questions. All questionnaires contained yes/no questions, questions on categorization with single and multiple choice, rating scales with an 11-point Likert scale and free text responses. On the 11-point Likert scale, 0 meant “not at all” and 10 meant “extremely”. Some of the questions in the questionnaire were open-ended and had to be completed using free text fields in order to avoid a bias by presenting preformulated answers only. These concerned the areas in which a variety of answers were to be expected, such as with regard to the reasons for seeking advice, the conflict situations experienced or the perceived effects or hurdles of CEC. Examples of this are “What do you consider to be problematic with regard to clinical ethics consultation? Are there any hurdles?” or “What positive effects have you noticed?”. The questionnaires were pretested (physicians, ethicists and nurses) and adapted according to the feedback.

For the survey, a letter was sent to all German perinatal centers (German perinatal centers (PNC) off level I and II N = 213) (The perinatal centers in Germany are categorized by the Levels of Care, but vice versa, to the international standard classification. PNCs with the highest level of care are classified as level I. In this regard, the following children are treated in level 1 perinatal centers (care level 1): premature infants with a birth weight of less than 1250 grams or with a gestational age of less than 29 + 0 weeks, triplets with a gestational age of less than 33 + 0 weeks and other multiples and infants with prenatally diagnosed fetal or maternal diseases where immediate specialized intensive medical care for the newborn is foreseeable after birth. This applies in particular to suspected congenital malformations (e.g., critical heart defects, diaphragmatic hernias, meningomyeloceles and gastroschisis)). One (questionnaire A, containing 5 questions) was addressed to the head physician of the pediatric department to evaluate the access and utilization of ethics consultation services and their assessment. The second (questionnaire B, containing 11 questions) was aimed at NICU heads (medical and nursing) who were asked about the use, assessment of usefulness and support by CEC.

As the survey was completely anonymous, it was neither possible to assign the respective questionnaires to individual centers, nor to identify which head of department had also completed questionnaire B. The in-house PNC in Marburg was not contacted and included in the study to avoid any form of bias. The survey period was July to October 2020. The anonymized data were analyzed using the methods of descriptive statistics and qualitative analysis of open text. All statistical evaluations were carried out using the IBM SPSS Statistics Version 26 program. T-tests were calculated for the questions to determine group differences with metric data. Although the data were not normally distributed, this test can be assumed to be sufficiently robust against the violation of the normal distribution assumption with a sample size of over 30 [26]. For the questions with the categorical data, cross-tabulations with Chi^2^-tests were calculated. Pearson correlations were calculated for the correlation questions. All calculations were performed two-sided with a set significance level of 5%.

The analysis of the open text passages was based on a qualitative content analysis according to Mayring [27]. Answers in the free text sections consisted partly of a string of keywords, but often also of several sentences. Parts or whole sentences of the written answers were first coded. The codes were grouped in a matrix and classified into similar topics. Afterwards the topics were subjected to overarching themes and assigned to comprehensive categories.

This study was reviewed by the Ethics Committee of the Department of Medicine of Philipps University, Marburg, Germany (corresponding code 98/18). Approval was given on 26 March 2019.

## 3. Results

### 3.1. Questionnaire A [Head Physicians of Pediatric Departments]

Questionnaire A was completed and returned by 81 head physicians of the department (response rate 38%). Every returned questionnaire could be included in the analysis, as no incomplete or unanswered questionnaires were returned.

**Figure 1:**
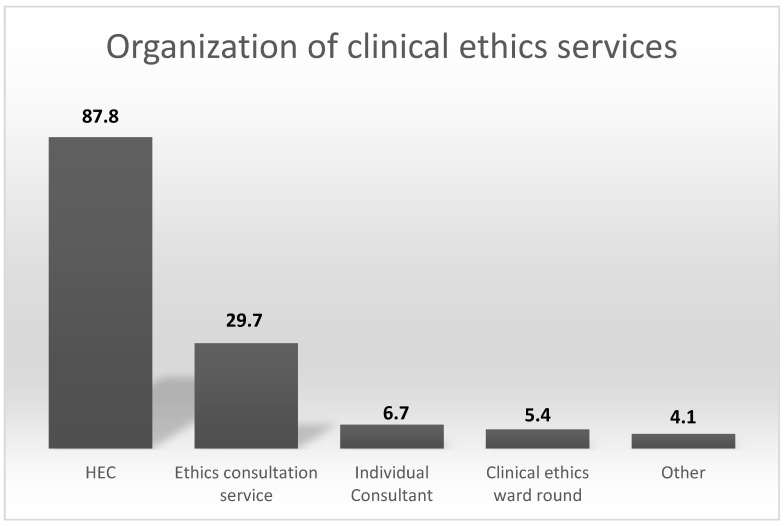
The organization of clinical ethics services.

Over ninety percent (91.35%) of the heads reported that ethics consultation services (CEC) were established in the department. Of these, the CEC are organized as hospital ethics committees (HEC) in 65 pediatric hospitals (87.8%), as a consultation service in 22 hospitals (29.7%) and as regular clinical ethics ward rounds or liaison services in 4 hospitals (see Figure 1). Nobody stated that she/he did not know the organizational structure of the CEC, although a corresponding answer option was specifically provided.

**Figure 2:**
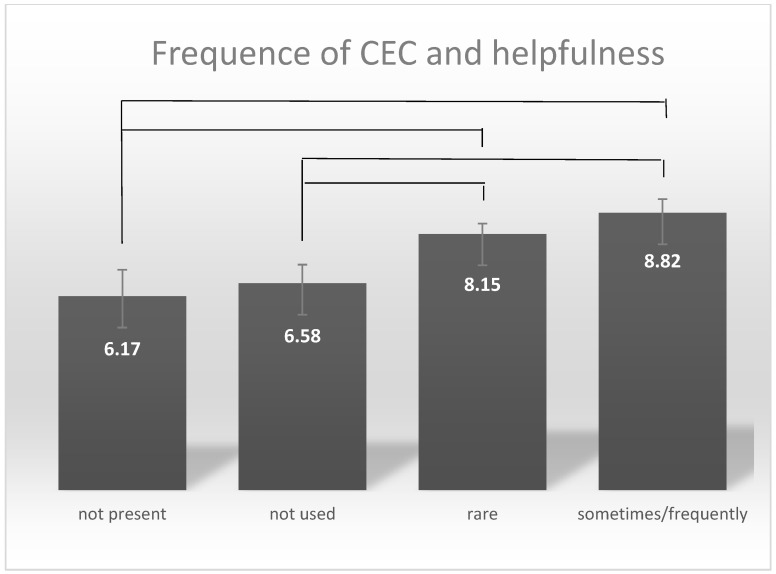
There is a statistically significant correlation between the frequency of use and the assessment as helpful. The numbers in the bars indicate the assessment of helpfulness in the respective frequency groups.

The heads of pediatric departments evaluated CEC as helpful (mean value 7.94 [Likert 0–10]. However, around half of respondents who stated that CEC were established reported that the service is predominantly rarely used (every 4–6 months, N = 40 (54.1%)). About one third make use of the CEC only occasionally or never (occasionally N = 19 (25.7%)/never N = 12 (16.2%)). There was a statistically highly significant difference between the frequency of the reported use of ethics consultation and the evaluation as helpful (the question was not explicitly aimed at the assessment of one’s own CEC: “How helpful do you think the instrument of clinical ethics consultation is in principle?”) (F (3.76) = 4.869, *p* = 0.004). If CEC were not available (MW 6.17; sd-error 0.833) or not used (MW 6.58, sd-error 0.589), CEC were rated as significantly less helpful compared to rarely (MW 8.15, sd-error 0.327, *p* = 0.029/*p* = 0.022), “occasionally” or often used CEC (MW 8.82, sd-error 0.425, *p* = 0.003/*p* = 0.006) (see Figure 2).

### 3.2. Questionnaire B [NICU Management]

Questionnaire B was addressed to the medical and nursing heads of the NICU. One hundred and twenty-eight completed questionnaires were returned. Of these, 20 people declared themselves to be “head of the department”, 52 people “nursing staff” and 56 people “consultant”. All questionnaires could be included in the analysis as there were no returns of incomplete or unanswered questionnaires.

The survey was designed as anonymous. Therefore, it was not possible to evaluate how many centers the participants belonged to. As we only provided the NICUs with two questionnaires each and asked the medical and nursing heads to participate in the cover letter, we can conclude—despite overlaps—that at least 52 (24.4%) and a maximum of 76 (35.7%) NICUs took part. A total of 94 people specified that they worked in a PNC of level 1, 20 people belonged to a PNC of level 2 and 14 people did not specify.

#### 3.2.1. Reasons for Consultation and Initiators

Free text responses concerning reasons for consultation were analyzed qualitatively. They could be assigned to three subject areas: (1) desired and structured reflection within the team in the event of prognostic certainty/uncertainty, (2) joint decision making and (3) (moral) dissent/moral conflicts. In terms of content, the questions mainly revolved around limiting therapy or switching to a palliative medical treatment concept for premature or full-term neonates with severe complications or complex malformation syndromes. Below are two typical answers from the free text fields:

“The conflict situations are similar. Discontinuation of therapy, divergent opinions of parents and team in severely disabled patients. However, I am of the opinion that we as practitioners (doctors/nurses), who know the patients/circumstances, must come to a decision, which we are always able to do at the end during the team meeting”.

“Determining treatment goals when the diagnosis is uncertain in or and disagreement between medical disciplines or doctors and nursing staff”.

One question asked who the initiators of the last five ethics consultations were (several answers were possible). The results are shown in Table 1.

#### 3.2.2. Evaluation and Positive Effects

**Figure 3:**
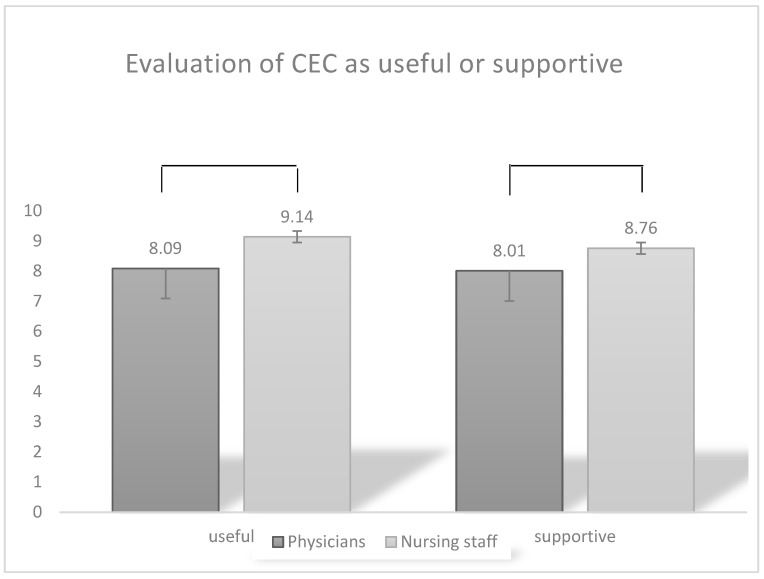
Participating professional groups rated CEC significantly differently in terms of their usefulness and support in everyday clinical practice.

The participants stated that they found the usefulness of the CEC and the support in everyday clinical practice to be very good (mean 8.52; 8.3, respectively [Likert 0–10]). Nursing staff rated CEC as significantly more useful (M = 9.14, SD = 1.06) than the physicians (M = 8.09, SD = 2.02) [t = −3.762, *p* < 0.001, Cohen’s d = 0.06] and felt more supported by the CEC (nursing staff M = 8.76, SD = 1.41; physicians (M = 8.01, SD = 1.98) [t = −2.298, *p* = 0.023, cohen’s d = 0.42] (See Figure 3).

Free text responses concerning positive effects were analyzed qualitatively according to content and categorized dichotomously as positive or not. The majority of all participants mentioned positive effects (75.0% heads of pediatric departments, 82.1% consultants and 76.9% nursing staff). Positive effects are summarized in Table 2. These could be subsumed under the following categories: (a) a reflected, structured decision in difficult situations, (b) achieving a shared decision with all professionals involved, (c) involvement of a “neutral instance”, (d) increased (legal) certainty and (e) emotional relief. While the benefits mentioned were similar within the professions, the frequency with which certain aspects were mentioned differed. While physicians cited the structured decision-making process and reflection on it as particularly important advantages of CEC, nurses most frequently mentioned the involvement of all professionals in the decision and the involvement of a “neutral” instance. Below are two answers concerning the positive effects:

“Offers the opportunity to communicate openly with all professional groups and also to *including the child’s point of view*”.

“Yes, the therapy regimes often become “clearer” for everyone involved and conflicts within the team are resolved; a uniform approach as a “team” can be better implemented”.

#### 3.2.3. Difficulties and Hurdles

Free text responses concerning difficulties and hurdles about CEC were analyzed qualitatively. The results are summarized in Table 2. Both internal dependence on hierarchies and the team members’ attitude towards ethics consultation and external reasons (logistical difficulties, lack of time, high emotional challenges or a lack of neonatology background knowledge of the consultants) were cited as barriers. As an example, there is the following answer concerning hurdles:

“Yes, time factor; difficult to assess in the case of imminent prematurity at the limit of viability; long pre-planning until ethics counseling is possible at our hospital. Long advance planning until ethics consultation is possible in our hospital, as several professional groups are involved”.

Logistical difficulties included a lack of time, time to prepare, staff shortages, language barriers with parents or legal concerns.

Unexpectedly, around one third of those who stated that they experienced hurdles addressed the particular content-related (and emotional) challenges for ethics consultants in neonatology: ethics consultants were considered of lacking “background knowledge” or experience in neonatology issues (field competence), leading to difficulties in understanding specific problems and conflicts in neonatology.

Beyond this, nursing staff accentuated team-related problems, such as hierarchies, that not all team members saw a need for discussion, that there was “resistance” from physicians or that physicians perceived ethics consultation as a form of criticism. For example, one person responded:

“In our hospital, the establishment of the clinical ethics committee has not had any effect on the PNC. The in-house committee (consisting of different specialist departments) does not help with neonatal questions/conflicts. There was often a lack of understanding for the special problems, so we developed our own procedure together with the obstetricians and the neonatologists from the cooperating university hospital.”

## 4. Discussion

The results of this study show that although CEC are well established in Germany, they are rarely used in neonatology. In some contrast to their underutilization, CEC are rated as very helpful and supportive overall. There was a significant difference in this assessment between physicians and nurses. Interestingly, in neonatology, the clinical field competence of ethical consultants seems to play a decisive role in the utilization of CEC.

The favorable evaluation of usefulness of CEC confirms previous data from the literature [28]. A number of studies have examined satisfaction as an outcome parameter for CEC [29,30,31,32], with different target groups such as patients, families and healthcare professionals included. The methods and evaluations differed in detail, with Likert scales often being used to express satisfaction with the process and outcome of the consultation. The majority of respondents reported satisfaction and rated the ethics consultation as helpful—two categories that do not necessarily correlate with each other, as Orr and colleagues have shown [30].

Most pediatric centers report 1–10 ethics consultation per year [33,34,35,36]. Only a very recent study evaluating pediatric ethics consultation services in the US report a frequency varying from 0–50 consultation in 12 months, whereby approximately 41% report a range of 0–10 consultations per year [37], similar to the present study. A study that compared the ethical challenges experienced in pediatrics with the number of ethics consultations requested concluded that formal pediatric ethical consultation services are underutilized, although more than eighty percent of staff members reported moral distress [38]. The fact that CEC are often underutilized, even though they are now largely well established, is a known problem in adult medicine [39]. Several reasons are known for this, such as perceived delays in clinical decision making, the feeling that they should solve the problems themselves (physicians should be the primary decision maker), a perceived lack of qualifications of the consultants or a lack of knowledge of the procedure [40,41]. Carter et al. have put forward a theory explaining underutilization in pediatrics CEC, according to which, a number of “moral spaces” exist in hospitals in which conversations about moral issues take place. They referred to Margaret Urban Walker’s description of ethicists as the architects of these moral spaces. They assume that a number of these moral spaces exist in the field of pediatrics and that many conversations take place on an informal level. Therefore, only a few situations lead to formal ethical consultations [36].

Instead, the results of this study indicate that barriers play a role. Of those named were logistical challenges, “hierarchies and concerns”. Particularly in the context of the known work overload and staff shortages, time–logistical requirements certainly contribute to lower utilization. It also appears that a lack of neonatological field expertise of the ethic consultants leads to restraint due to concerns that this makes the problems more incomprehensible. Lilje already problematized the issue of ethics consultants’ qualification almost 30 years ago: “In any case, CECs require sufficient expertise in both disciplines: in applied ethics and in clinical medicine”. However, he directly qualified: “Of course, a real double training would be ideal, although in practice it can hardly be realized” [42]. Since then, this problem has been repeatedly discussed and a solution sought. In fact, patient care in neonatology is specialized. It is well known that confrontation with ethical problems is particularly high in pediatrics, especially in neonatological intensive care [1,2,3,4]. In the light of “moral spaces”, it must be considered that clinical ethicists should contribute to creating this space. A lack of field expertise on the part of consultants, particularly in the specialized field of neonatology, could give rise to the fear that the special situation of infants will not be easily understood by the consultants and thus hinder the request of ethics consultations.

In addition, however, the experience gained with ethics consultations also appears to be a factor in their utilization, pointed out by the significant correlation between the frequency of use and the assessment of clinical ethics consultation “as helpful”. It is known that knowledge about and experience with ethics consultations lead to significantly more requests [40,43]. This is particularly the case if the experience is positive [44]. It, therefore, seems to be a vicious circle. The positive correlation between ethics consultation and the quality of patient care has already been demonstrated [16,20], as has the reduction in moral stress, which leads to a healthier climate among ward staff [45]. If underutilization is related to the barriers mentioned, positive experiences that could lead to more use of CEC will not be made. Since moral distress is also very high in neonatology, this means that an opportunity to counter this effectively is lost [45,46,47,48,49,50]. This may be particularly applicable for the nursing staff and it is also supported by this study.

In the present study, similar to others, the assessment of CEC by nursing staff differs significantly from that of physicians [11,31,51,52,53,54,55]. Nurses find ethics consultations more helpful. This varying valuation between physicians and nursing staff can be attributed to differences in training, the focus on patient care and direct contact with patients. In neonatology in particular, the proximity of nursing staff to their patients can lead to higher moral distress, as nurses experience the concerns of the families and deal more intensively with the needs of the children [46,53]. Staying with the consideration of “moral spaces”, it seems to be particularly important for the nurses to extend this to all professions involved, considering that they emphasized this point particularly in the benefits they experienced.

In view of the feedback that CEC are regarded as very helpful and useful, but are obviously underused, hurdles should be taken into account, in particular, possible hierarchies in the medical team and logistical challenges. The organization of ethics consultations is also rather complicated and quite time-consuming. On the other hand, in addition to medical statistics and guidelines for the care of extremely immature infants, ethical training for all those involved in the process is essential to create more and maintain “moral spaces” and lead to more confidence in the team. In this respect, however, it may also be helpful to offer ethics consultants in Germany special training for counseling in neonatology. Carter’s working group also proposed discussing ethical dilemmas at various points in the pediatric hospital and further developing clinical ethics consultation services [36]. It is, therefore, worth considering whether a different logistical approach would be preferable. The logistical requirements should be as low as possible, but the medical team should be able to discuss and reflect on ethical problems and receive training in medical ethics. Therefore, an alternative approach would be an ethics liaison service or clinical ethics ward rounds. In practice, liaison services or clinical ethics ward rounds based on the “*Marburg model*” are suggested here as low-threshold offers for the entire medical team [44,56,57]. There are two different forms of preventive ethics counseling. In the liaison service, an ethicist accompanies the regular ward round; in a clinical ethics ward round, an additional, regular ward round is set up with an ethicist. This way of preventive ethics consultation helps to strengthen the team through social-communicative processes as well as the ethical competence of its members and enables them to find independent solutions. Nevertheless, the “view seen from outside” provided by the ethics consultation was appreciated and the protected atmosphere in the familiar setting in regular ward rounds and discussions was perceived as positive [57]. Therefore, both approaches have in common that a regular “moral space” is created; it is planned into the daily routine and so logistical hurdles are lower. In this way, sensitivity for ethical challenges in the medical team and the field experience of the ethics consultants could be improved [58,59].

Limitations: This is a quantitative questionnaire survey, which is subject to the usual limitations of quantitative evaluation studies. These include the response rate, the implicit assumptions in the questionnaire design, the response behavior, which may be subject to a “socially desirable” bias, and, of course, the selection bias that always arises in questionnaire studies due to the fact that only people who are interested in the topic or, in this case, feel professionally ethically committed respond to questionnaires. The response rate is low (24–38%), meaning that the data collected cannot be generalized without further ado. In summary, it should be noted that no general conclusions could be drawn and no real dependencies could be determined or proven, particularly due to the study design. At the time of the survey, there were no standardized competence levels for ethics consultants, so we did not ask about the qualifications of ethics consultation members. Our survey was also limited to the users of ethics consultations and not the providers. Particularly in view of the argument that ethics consultants lack expertise and are emotionally burdened, the survey would have been enriched by the assessment of people who provide ethics consultation.

Nevertheless, this study provides an initial and fairly complete overview of the establishment and use of clinical ethics consultations in German neonatal intensive care units and the assessment of this by the users.

## 5. Conclusions

Overall, the results of the study suggest that ethics consultations in neonatology should take account of the special situation in this area by considering possible, specific barriers in order to counteract underutilization. This could be achieved, for example, through a model of preventive ethics counseling. However, these approaches to ethics consultation still require further research, particularly in the field of pediatrics and neonatology.

## Figures and Tables

**Table 1:** Initiators of the last 5 ethics consultations (multiple answers were possible, 103 persons with 266 responses).

Initiators	N	Profession
head physician	35	“physician”
consultant	77	
physician staff	24	
physician other than pediatrics	5	
nursing head	47	“nurses”
nursing staff	67	
pastoral care	2	“Other”
parents	2	
not specified or “other”	30	

**Table 2:** Summary of the observed positive effects of ethics consultations and difficulties/hurdles for ethics consultation services (the order does not include any ranking).

**Positive Effects**
reflected, structured decision in difficult situations
achieving a shared decision with all professions involved
involvement of a “neutral instance”
increased (legal) certainty
emotional relief
**Difficulties/Hurdles**
internal dependence on hierarchies
“resistance” from physicians
team members’ attitude towards ethics consultation
logistical difficulties (lack of time, staff shortages…)
lack of neonatology field competence of ethic consultants

## Data Availability

The data collected are included in the article; the underlying raw data are not published for use in further research.
